# Upcycling Rocha do Oeste Pear Pomace as a Sustainable Food Ingredient: Composition, Rheological Behavior and Microstructure Alone and Combined with Yeast Protein Extract

**DOI:** 10.3390/molecules28010179

**Published:** 2022-12-25

**Authors:** Ana Fernandes, Sara Simões, Isabel M. P. L. V. O. Ferreira, Maria João Alegria, Nuno Mateus, Anabela Raymundo, Victor de Freitas

**Affiliations:** 1 LAQV/REQUIMTE, Chemistry and Biochemistry Department, Science Faculty, Porto University, 4169-007 Porto, Portugal; 2 LEAF—Linking Landscape, Environment, Agriculture and Food Research Centre, Instituto Superior de Agronomia, Lisbon University, 1349-017 Lisboa, Portugal; 3 LAQV/REQUIMTE, Laboratory of Bromatology and Hydrology, Department of Chemical Sciences, Faculty of Pharmacy, Porto University, 4050-313 Porto, Portugal; 4 SUMOL + COMPAL Marcas S.A., 2790-179 Carnaxide, Portugal

**Keywords:** bioactive compounds, pear pomace, phenolic compounds, yeast protein extract (YPE), rheological behavior, texture, viscosity

## Abstract

This work explores the potential of Rocha do Oeste pear pomace to be used as a sustainable and healthy food ingredient. Moreover, the enrichment with yeast protein extract (YPE) may be useful to design innovative food products. The main goals of this study were to assess pear pomace concerning: (i) chemical composition and antioxidant capacity; (ii) rheology, texture, and microstructure characterization (alone or enriched with YPE), before and after heating. The results showed that pear pomace was a rich source of dietary fibers (74.5% DW), with phenolic compounds (3.9 mg chlorogenic acid equivalents/g dry weight), also presenting antiradical activity (3.90 μmol Trolox equivalents/g DW). Pear pomace showed a shear thinning behavior and a typical soft-gel behavior, which was not affected by YPE enrichment, thus suggesting that YPE did not affect pear pomace technological properties. Thermal treatment also did not alter pear pomace rheological properties. YPE addition induced a decrease in the apparent viscosity and a destabilizing effect, compared to the samples that were subjected to thermal processing. These results highlight the importance of pear pomace and the use of YPE for protein enrichment, opening new opportunities for their exploitation.

## 1. Introduction

Rocha do Oeste Pear (*Pyrus communis* L.) is a Protected Designation of Origin (PDO) variety of pear and one of the most relevant fruits in Portugal. It is distinguished by its firmness, acidity, soluble solids content, color, as well as high digestibility, nutritional value, and high content of antioxidant phenolic compounds [1,2,3]. Pear juice pilot scale processing by SUMOL + COMPAL, an important Portuguese fruit juice company, encompasses a centrifugation process of the fruit puree, to produce a turbid juice. Pulps centrifugation results in considerable amount of pomace (skins, seeds and flesh), mainly constituted of sugars, fibers, pectins, and other insoluble carbohydrates, minerals, organic acids and phenolic compounds [1]. Due to its composition, pear pomace may be applied as a cost-effective source of bioactive and functional compounds [2], as a food ingredient to control food texture and rheology behavior or be converted as a functional flour [4,5], thus upcycling food industry side streams.

Phenolic compounds are one of the most targeted compounds from fruit co-products due to their biological properties and technological role as food additives and functional supplements [6]. Regarding pear pomace, there is little data available in the literature related to these compounds. However, it can be expected that the same bioactive compounds present in the fruit are present in its bio-residues. In terms of total extractable phenolic compounds, Rocha do Oeste puree (peel and flesh) showed to be composed of chlorogenic acid, syringic, ferulic and coumaric acids, arbutin and (-)-epicatechin as the major components [7].

Dietary fibers can also find a wide application in the food industry, holding high promise as a potential food additive and/or as a functional food ingredient due to their functional properties, including water-holding capacity, gel-forming, fat mimetic, texturizing, and thickening properties. Additionally, dietary fibers are regarded as one of the most effective ways to prevent and control chronic diseases caused by over-intake of fat, since they contribute to satiation, reduction of pre-prandial cholesterol and postprandial blood glucose levels [8], obesity control [9], digestive health [10], immunity response and intestinal mucosa integrity [1]. Total dietary fiber content in pear pomace from different cultivars was found to be significantly high, varying from 44 to 79% on a dry weight basis [4,5,11], with insoluble dietary fibers isolated from pear pomace, preventing high-fat diet-induced obesity in rats mainly by improving the structure of the gut microbiota [12]. Phenolic compounds and dietary fibers are generally studied separately due to differences in their chemical structures and physicochemical and biological properties. However, dietary fibers with associated phenolic compounds have become increasingly interesting as they could be useful for the food industry to enhance the bioactive and technological properties of the products as it combines the properties of both components in a single material [13].

Despite the rich nutritional composition of pear pomace, its protein content is poor. Yeast protein extracts (YPEs) can be used in combination with fruit pomaces in the development of innovative food products. The use of YPE as a food ingredient is aligned with the increasing demand of alternative protein sources, besides plants and meat, being suitable for vegan, vegetarian, and flexitarian diets. Due to its content on easily digestible proteins with a balanced amino acid composition, trace minerals, vitamins including B-group, and antioxidants, yeast protein is a versatile ingredient in different preparations as health supplements and natural flavor compounds for the food industry [14]. The most widely studied YPEs were obtained from *Saccharomyces cerevisiae* cell wall, although there are many variants of YPE which can be obtained from other components of yeasts, including cytoplasm and vacuole [15]. In this work, for pear pomace enrichment, a commercial cytoplasmatic YPE was used [16].

The study presented herein is part of cLabel+ project, and it intends to add-value to pear pomace, a co-product generated from the production of pear turbid juices, in a circular economy rationale. Therefore, the main goals were to characterize pear pomace nutritional and phytochemical profile, and its antioxidant capacity. Additionally, as pear pomace incorporation may have an impact on the food structure, flow and linear viscoelastic behavior, texture properties and microstructure of the pear pomace were also studied. The effect of YPE addition (increasing pear pomace protein content) and thermal processing (pear pomace alone and combined with YPE) on pear pomace rheological, textural and microstructure properties was also assessed. With this, it is aimed to upcycle pear pomace into the food value chain, thus creating valuable food ingredients with technological, functional and health benefits.

## 2. Materials and Methods

### 2.1. Reagents

All reagents and solvents used were of analytical grade. Water was purified with a Milli-Q system (Millipore, Bedford, MA, USA). Folin-Ciocalteau reagent, Trolox, (+)-catechin and chlorogenic acid were obtained from Sigma-Aldrich. Yeast protein extract (YPE) from *Saccharomyces cerevisiae*, Divino^®^ was provided by Proenol, a biotechnological industry [16,17]. YPE was composed of proteins and carbohydrates (70:30, *w:w*). YPE protein content and characterization is reported in the literature [18].

### 2.2. Pear Pomace Processing

Pear pomace, provided by Sumol + Compal, was obtained as a co-product from pear juice production at pilot scale, through pear puree centrifugation. After defrosting at 4 °C for 24 h, pear pomace was washed with water to remove free sugars and other soluble nutrients (4 L of water per 1 kg pomace), allowing a broader application of this fruit flour into non-sweet food products. Cold water was used in the washing process to avoid starch dissolution. Pear pomace was soaked in water and stirred for 5 min, filtered on a filter cloth and manually pressed to drain excess water [19]. The residue was frozen and freeze-dried for at least 48 h. A domestic grinder was used to obtain a homogeneous pear pomace flour (PPF) after freeze-drying. The flour was then packed air-tight in plastic bags and stored at −20 °C for further use.

### 2.3. Proximal Composition Analysis of Pear Pomace

The proximal composition of dried pear pomace (moisture, ash, protein, fat, dietary fibers, and carbohydrates) was determined according to standard AOAC methods. Moisture was determined by weight loss after 12 h drying in a drying oven, at 105 °C. Ash was determined on a muffle furnace. Fat was assessed by Soxhlet, while protein content was determined by the Kjeldahl method. Total dietary fibers (TDF) and insoluble dietary fibers (IDF) were determined according to the enzymatic-gravimetric method using a total dietary fiber kit (Megazyme Assay Kit, Wicklow Ireland), based on AOAC Method 991.43 [20]. The remaining difference to 100%, was comprised of non-DF carbohydrates. All determinations were performed in duplicate.

### 2.4. Phenolic Composition of Pear Pomace

The extraction of phenolic compounds was performed according to an adaptation of the methods previously described [21,22]. Hydroalcoholic solution and acetone/water mixture were used to extract free phenolics, after which the residue was subjected to a sequential base and acid hydrolysis to recover bound phenolics. 0.5 g of freeze-dried pear pomace was mixed with 20 mL water:methanol (50:50, *v*/*v*, pH 2) and then ultra-turraxed at 9500 min^−1^ for 10 min (T25-Ultra-turrax, IKA-Labortechnik^®,^ Staufen, Germany). The extract was centrifuged for 15 min at 4 °C and 10,000 rpm (Dynamica Velocity 14 R Refrigerated Centrifuge, Dynamic Scientific Ltd.; Livingston, UK) and the residue was re-extracted with 20 mL of acetone/water (70:30, *v*/*v*), as described above. The supernatants of each extraction were combined and evaporated to dryness under vacuum at 37 °C on a rotary vacuum evaporator, and redissolved in 2.0 mL methanol, giving an extractable phenolic fraction (**EF**). The remaining residue was subjected to a sequential base and acid hydrolysis according to the methods previously described [23,24]. For base hydrolysis, the residue resulting from the extractable phenolic fraction, was hydrolyzed for 4 h with 15 mL of 2M NaOH at room temperature, under a stream of nitrogen. The resulting slurry was acidified to pH 2 with HCl (6M) and then centrifuged for 15 min at 4 °C and 10,000 rpm. The supernatant was extracted with diethyl ether/ethyl acetate (DE/EA) (1:1, *v*/*v*) three times. The combined organic fractions were evaporated to dryness and subsequently dissolved in 4.0 mL methanol, giving a base-hydrolyzable non-extractable phenolics (**BNEF**). For the subsequent acid hydrolysis, the remaining residue after base hydrolysis was incubated with 15 mL of 2 M HCl, heated at 85 °C for 1 h, brought to pH 2 with 5 M NaOH and then treated, as described above, giving an acid-hydrolyzable non-extractable phenolics (**ANEF**).

### 2.5. Determination of Total Phenolic Content (TPC)

Total phenolic content was estimated through an adaptation of the Folin-Ciocalteau colorimetric method [25]. The reaction mixture was prepared by mixing 15 μL of each extract (or water for control) with 75 μL of Folin-Ciocalteau phenol reagent and 500 μL of distilled water in 2 mL Eppendorf tubes. The mixture was vortexed for 10 s, followed by the addition of 300 μL of 5% Na_2_CO_3_. The mixture was brought up to 1500 μL by adding 610 μL of distilled water and vortexed for another 10 s. After 30 min incubation at room temperature in the dark, the absorbance was measured at 750 nm. Results were expressed as mg chlorogenic acid equivalents per g of dry weight (mg CAE/g DW).

### 2.6. Phenolic Compounds Analysis

Phenolic compounds quantitative analysis was performed by HPLC-DAD [26]. Chromatography was carried out on an Elite LaChrom Merck Hitachi, composed of the following modules: quaternary pump (L-2130), auto-sampler (L-2200), thermostated column compartment (L2300) with a Purosphere^®^ STAR Lichrocart, C-18 reverse-phase column (150 mm × 4.6 mm, i.d.; 5 μm), thermostated at 25 °C and a diode array detector (L-2455). The mobile phase consisted of COOH/H_2_O (1/99, *v*/*v*) (solvent A) and CH_3_CN (solvent B). The flow rate was 0.5 mL.min^−1^ with a linear gradient ranging from 97% A to 18% B in 45 min, then reaching 100% B in 5 min, a final isocratic gradient of 100% B during 7 min and a final re-equilibration isocratic gradient of 97% A for 5 min. Spectra were recorded between 220 to 600 nm and detection was carried out at 280 nm and 320 nm as the preferred wavelengths. The concentration of the identified polyphenolic compounds was expressed in chlorogenic acid equivalents (CAE) or catechin equivalents (CE) per g DW.

Identification of extractable phenolic compounds (**EF**) was performed by LC-DAD/ESI-MS using the same solvents and gradient, as previously described. A Finnigan Surveyor series liquid chromatograph equipped with a Thermo Finnigan (Hypersyl Gold) C-18 reversed-phase column (150 mm × 4.6 mm, i.d.; 5 μm, Thermo Scientific, Waltham, Massachusetts, USA), thermostated at 25 °C was used. Detection was carried out between 200 and 700 nm using a Finnigan Surveyor PDA Plus detector. Mass detection was made on a Finnigan LCQ DECA XP MAX (Finnigan Cor., San Jose, CA, USA) quadrupole ion trap equipped with an atmospheric pressure ionization (API) source using an electrospray ionization (ESI) source. The vaporizer and capillary voltages were 5 kV and 4 V, respectively. The capillary temperature was set at 325 °C. Nitrogen was used as both sheath and auxiliary gas flow rates of 80 and 30, respectively (in arbitrary units). Spectra were recorded in the negative- or positive-ion mode between *m*/*z* 120 and 2000. The mass spectrometer was programmed to do a series of three scans: a full mass, a zoom scan of the most intense ion in the first scan, and a MS-MS of the most intense ion using relative collision energies of 30 and 60 V.

### 2.7. Antioxidant Assays

Antioxidant activity was measured by two different assays, namely ferric reducing antioxidant power (FRAP) and free radical scavenging activity (DPPH), according to the literature [27,28]. In 96-well plates, 270 µL of diluted FRAP reagent (10 volumes of 300 mM acetate buffer, pH 3.6 + 1 volume of 10 mM tripyridyltriazine (in 40 mM HCl) and 1 volume of 20 mM FeCl_3_) at 37 °C and 30 µL of each fraction were mixed (in triplicate). Absorbance increase was measured at 593 nm at 0 and 4 min. For DPPH assay, 270 µL of DPPH solution (2,2-diphenyl-1-picrylhydrazyl prepared in methanol at 60 µM) was mixed with 30 µL of each fraction (in triplicate). Absorbance decrease was recorded at 515 nm every 5 min, for 20 min. Results were calculated in % Radical Scavenging Activity (%RSA) and inserted in the calibration curve. For both methods, control samples were prepared with methanol (DPPH) or Milli-Q water (FRAP), and absorbances were measured on a plate reader (*Biotek Powerwave XS,* Santa Clara, California, USA). Results were expressed as μmol Trolox equivalents per g DW. QUENCHER procedure was also applied to assess the direct antiradical activity of pear pomace, without any previous extraction steps, according to an adaptation of the methodology described elsewhere [29,30]. A total of 2.0 mg of pear pomace was mixed with 1.7 mL of DPPH reagent. The mixture was left under agitation for 20 min, to facilitate the surface reaction between the pear pomace insoluble material and the free radical. After this period, samples were centrifuged (1 min at 13,400 rpm) and the absorbance of the optically clear supernatant was directly measured at 515 nm. Results were expressed as μmol Trolox equivalents per g DW.

### 2.8. Preparation of Pear Pomace Samples for Rheological Measurements and Texture Analysis

Samples were prepared by dispersing 7.5 g of pear pomace, (PP-Control) or pear pomace enriched with yeast protein extract (10% *w*/*w*) (PP-YPE-Mixture) in distilled water until the weight of the pastes reached 22.5 g. The resulting pastes were shaped into balls and maintained at 4 °C for 12 h. Each formulation was prepared in sextuplicate (Table 1). Three samples of each Control and 10%YPE-enriched pomace were subjected to a thermal treatment to promote thermal gelation yielding heated pear pomace (H-PP), and heated pear pomace enriched with YPE (H-PP-YPE). Samples were placed on a water bath at 77 °C. When the samples reached 70 °C, heating was kept for an additional 10 min. After heating, formulations were kept at 4 °C for at least 12 h. Prior to texture and rheology evaluation, the resulting pastes were left at room temperature for 1 h.

### 2.9. Rheological Measurements

Rheological measurements (flow behavior and small amplitude oscillatory shear properties) were carried out on a controlled stress rheometer (Haake Mars III–Thermo Scientific, Waltham, MA, USA), coupled with an UTC-Peltier system, Haake Mars III Controller and air compression system Eheim Professional, according to the methodology previously described [31,32]. This included both stress sweep and frequency sweep tests, temperature ramp tests and flow curves. Four samples were evaluated, namely pear pomace (PP-Control), pear pomace enriched with YPE (PP-YPE), heated pear pomace (H-PP), and heated pear pomace enriched with YPE (H-PP-YPE). Samples were analyzed using a serrated parallel-plate geometry sensor with a 20 mm diameter (PP20) and a 2 mm gap (previously optimized for this type of paste). After placing the rheometer in the measuring position, the samples’ edges were coated with liquid paraffin to prevent moisture losses during tests. All samples were maintained in the sensor system for 10 min before running any rheological test, to reach a steady stable temperature and structure. For each sample, the linear viscoelastic region (LVR) was determined prior to the frequency sweep, at 6.28 rad/s (1 Hz) at 20 °C, through a stress sweep test. The viscoelastic properties of the paste were determined from the frequency sweep test, performed at the LVR of each sample, over a frequency range of 0.01–100 Hz or 0.001–100 Hz. Storage (G′) and loss (G′′) moduli (Pa) data *vs* angular frequency ω (rad/s) were fitted through power-law equations.

The viscoelastic properties of the non-heated pear pomace (PP) and pear pomace-yeast protein extract (PP-YPE) samples were also determined under four different time and temperature ramp experiments, within samples’ LVR [31,32]. Each sample was heated from 20 to 75 °C at 5 °C/min heating rate (step 1). After reaching this temperature, samples were kept at 75 °C for 10 min (step 2). Next, the samples were subjected to cooling until reaching 5 °C, at 5 °C/min cooling rate (step 3), being kept at these conditions for another 30 min. The time and temperature ramps were acquired at a constant frequency (1 Hz). Finally, a frequency sweep test was performed over a frequency range of 0.01–100 Hz.

Flow curves were obtained in triplicate, using serrate parallel plates with 20 mm of diameter (PP20) with a 2 mm gap, at 20 °C, with shear rates ranging between 1 × 10^−8^ and 1000 s^−1^, stepping up every 33 s to ensure the steady-state on each shear rate. The obtained curves were adjusted to the Williamson model (Equation (1)) using Origin 2019b (9.65) (OriginLab) software:

(1) $$\eta =\frac{\eta_{0}}{1+{\left(k\dot{\gamma }\right)}^{m}}$$

where η_0_ is the zero-shear Newtonian viscosity (Pa.s), k the consistency coefficient, and m is a dimensionless shear-thinning index. All rheology measurements were repeated at least three times.

### 2.10. Texture Profile Analysis

Texture profile analysis (TPA) was performed using a Texture Analyser texturometer (TA.XT plus Texture Analyser, Stable MicrosystemGodalming, UK), equipped with a 5 kg load cell, using a 11 mm diameter cylindric probe, according to an adaptation of the methodology [32]. Prior to analysis, samples were left to stabilize at room temperature for at least 1 h and placed in a 3 cm diameter cylindrical bottle (4 cm height). Texture analysis conditions included: pre-test speed 2.0 mm/s, post-test speed 2.0 mm/s, test speed 1 mm/s and distance of 8.0 mm. The calculation of TPA parameters was obtained by graphing a curve using force *vs* time plots, determining the values of firmness, cohesiveness and adhesiveness. Measurements were repeated at least six times for each sample.

### 2.11. Water Activity

Water activity (a_W_) of the pastes was determined using a thermo-hygrometer (HygroPalm HP23-AW, Rotonic AG) at 20 ± 1 °C. The a_W_ determination was performed in duplicate for the Control, H-PP, PP-YPE and H-PP-YPE.

### 2.12. Microstructure of the Pear Pomace

A microstructural analysis of the prepared pastes (surface) was performed by SEM/EDS using a High Resolution (Schottky) Environmental Scanning Electron Microscope with X-Ray Microanalysis and Electron Backscattered Diffraction analysis: FEI Quanta 400 FEG ESEM/EDAX Genesis X4M. Each sample was mounted and sputter-coated with a thin layer of gold–palladium alloy using the SPI Module Sputter Coater equipment. Digital images were collected at an accelerating voltage of 5–10 kV. Samples were observed with 100-, 500-, and 1000-fold magnification.

### 2.13. Statistical Analysis

Experiments were performed at least 3 times for each experimental condition (prepared in triplicate) to ensure the reproducibility of the results. Data was expressed as the mean ± standard deviation (SD). One-way analysis of variance (one-way ANOVA) was used to determine statistically significant differences between the means of different experimental groups, using the Tukey’s multiple comparisons test. Differences were statistically significant at *p* < 0.05. All statistical data were processed using GraphPad Prism version 8.0 for Windows.

## 3. Results and Discussion

### 3.1. Proximate Composition of Pear Pomace

The proximate composition (moisture, protein, ash, fat, and dietary fibers) of the pear pomace flour is depicted on Table 2. The PP flour was composed mainly of dietary fibers (TDF, 74.5%), with small amounts of protein (1.8%, DW), fat and ash (both 1.1%), on a dry weight basis. Moisture accounted for 6.3%. Insoluble dietary fibers (IDF) are the largest fraction, contributing approximately to 61% of pomace dry weight, while soluble dietary fibers (SDF) represent 13.4%. This result is within the range obtained in the literature [4,5,11], indicating a larger proportion of insoluble dietary fibers in pear pomace, although higher than those reported for “Rocha” pear pomace [1]. The differences in these values may be related to the maturation stage of the harvest, but also due to the washing procedure applied to the pomace to remove simple sugars. Products with a fiber content greater than 50% TDF are considered a rich source of dietary fiber [33]. Moreover, the SDF:IDF ratio is also important for the dietary and functional properties of fibers as both fractions are complementary, related to their functional properties. It is considered that the best ratios to achieve both health and technological properties of dietary fibers, include 30–50% of SDF and 70–50% of IDF [34]. For pear pomace, this ratio is close to 1:4 (SDF:IDF). In this respect, pear pomace does not appear to have a good balance. In previous studies, fruit and vegetable pomaces with slightly higher IDF content and slightly lower SDF content resulted in higher water absorption capacity and different results in the texture and rheological properties of the final products [35]. In fact, IDF is more useful for stabilizing and texturizing purposes, such as minimizing shrinkage, retarding staling, controlling moisture and increasing food stability [36].

### 3.2. Phenolic Compounds of Pear Pomace

**Figure 1:**
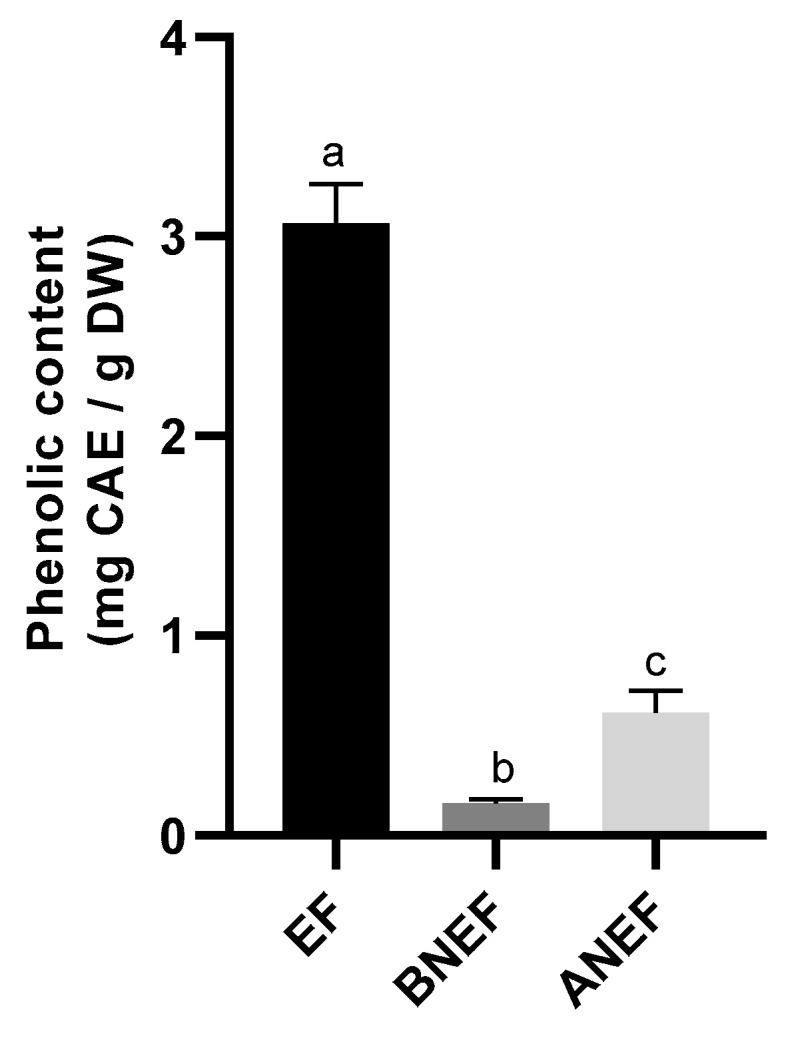
Extractable (EF) and non-extractable phenolic content (BNEF and ANEF) of pear pomace. Columns with different letters are statistically different (*p* < 0.05).

In fruit and vegetables, phenolic compounds can occur as extractable, soluble extractable conjugates (e.g., glycosides, esters of fatty acids) and non-extractable forms. The latter can be either chemically linked through ester, ether, or glycosidic bonds, to cell wall structural components, such as polysaccharides or proteins, or be physically entrapped in food matrixes and intact cells [37,38,39]. In this work, total phenolic content in pear pomace, analyzed by the Folin-Ciocalteau assay, was 3.9 mg CAE/g DW pomace (extractable + non-extractable phenolics), with free phenolics corresponding to 3.1 ± 0.2 mg CAE/g DW (Figure 1). This value was contained in the range of those presented in the literature [40] for different pear cultivars’ pomaces, but slightly lower than that obtained for “Rocha” pear pomace [1]. This suggests that pomace pre-washing to remove small sugars, may have caused a loss in soluble phenolic compounds. The lower phenolics levels in the pomace, compared to the mentioned study, can also result from the different ripening index or campaign year. The non-extractable phenolics in the remaining residue after aqueous/organic extraction accounted for approximately 20% of the total phenolic compounds in pear pomace. These values were in agreement with those reported in the literature, indicating that bound phenolics comprise an average of 24% of total phenolics in fruits and vegetables [41]. These results also showed that most of the non-extractable phenolics were recovered through acid hydrolysis (0.6 ± 0.1 mg CAE/g DW), compared to those recovered from base hydrolysis (0.16 ± 0.02 mg CAE/g DW) indicating a much more efficient liberation of phenolic compounds through acid hydrolysis, through the cleavage of ester and glycosidic bonds. For apple pomace bound phenolics, an opposite behavior could be observed with a higher content of phenolic compounds being released through base hydrolysis [21].

The quantitative and qualitative characterization of the extractable phenolic compounds was also performed by HPLD-DAD/ESI-MS analysis (Figure S1 and Table S1). A total of 12 phenolic substances were identified based on mass parent ion (*m*/*z*) and secondary (MS^2^ and MS^3^) fragment ions data. Chlorogenic acid was the predominant phenolic compound (16.3 mg CAE/100 g DW). (-)-epicatechin (7.1 mg CAT/100 g DW and procyanidins (dimeric and trimeric procyanidins, 5.5 and 2.3 mg CAT/100 g DW, respectively) were also present, together with quinic acid (3.0 mg CAE/100 g DW) and arbutin (3.9 mg CAE/100 g DW). Flavonols, such as quercetin-3-*O*-rutinoside (2.1 mg CAE/100 g DW), quercetin-3-*O*-galactoside (1.3 mg CAE/100 g DW), quercetin-3-*O*-glucoside (2.1 mg CAE/100 g DW) and isorhamnetin-3-*O*-rutinoside (1.5 mg CAE/100 g DW) accounted for approximately 19% of total phenolic compounds.

### 3.3. Antioxidant Activity

**Figure 2:**
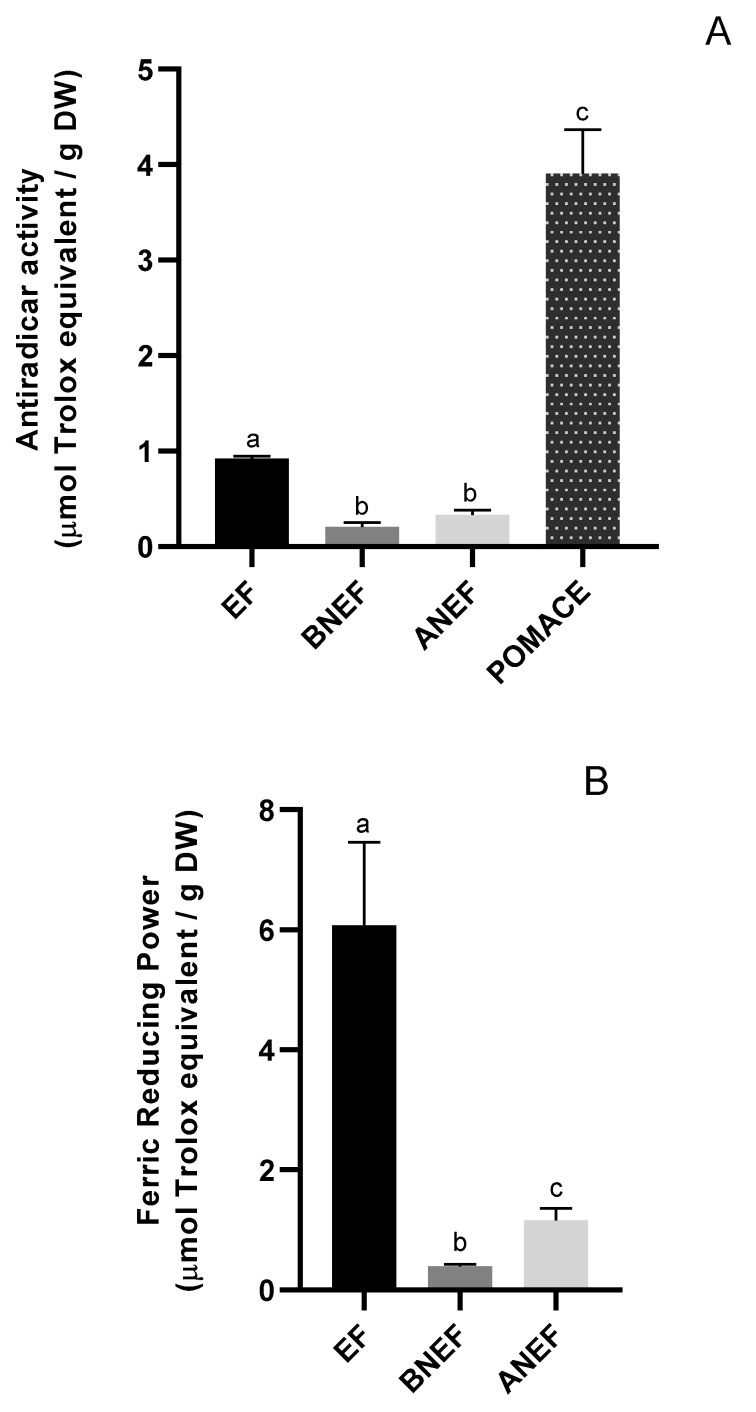
(**A**)-Antiradical activity of extractable (EF) and non-extractable fractions (BNEF and ANEF), together with insoluble pear pomace. (**B**)-ferric reducing power of extractable (EF) and non-extractable fractions (BNEF and ANEF). Columns with different letters are statistically different (*p* < 0.05).

Regarding the antioxidant capacity, for both FRAP and DPPH assays, a significantly higher antiradical capacity (*p* < 0.05) was observed for the extractable phenolic compounds fraction, correlated to its superior content in phenolic compounds (Figure 2). The non-extractable fractions (acid and base hydrolyzed) evidenced a similar antiradical capacity (*p* > 0.05), despite the higher amount of phenolic compounds recovered in the acid-hydrolyzed fraction. Regarding the reducing power, the acid hydrolyzed fraction showed a higher capability (*p* < 0.05). Although presenting lower antioxidant capacity compared to the soluble phenolics these results showed that not only extractable but also non-extractable fractions of pear pomace (alkaline and acid released), had good potential for valorization as antioxidants, contributing with 20 to 38% to the total antioxidant capacity (FRAP and DPPH assay, respectively). In fact, due to its macromolecular nature, non-extractable phenolic, connected to polymeric cell wall material may be released for absorption by intestinal microbiota in vivo and contribute to the overall beneficial activities and exert antioxidant activity through a surface reaction [42].

The antiradical activity of insoluble pear pomace was determined through the QUENCHER procedure. Using this approach, the soluble moiety of the pear pomace may exert its antioxidant capacity by quenching the free DPPH radical in solution, according to the usual liquid–liquid type reaction, while at the same time, the insoluble part may exert their antioxidant activity in the solid–liquid interface [30]. Results showed an antiradical activity significantly higher for the insoluble pear pomace (direct procedure), compared to the sum of the antiradical activity of the sequential extraction (hydroalcoholic extraction plus alkaline and acid hydrolysis, 3.90 *vs* 0.92 mmol Trolox equivalents/g DW). From this result, it can be suggested that both alkali and acid pre-treatment did not allow the complete liberation of bound antioxidants or that they were lost during the extreme conditions used in the hydrolysis procedure. On the other hand, the loss of interactions between antioxidants may have reduced the possible synergistic effects. In the case of cereals, the total antioxidant capacity (assessed by the ABTS methodology) obtained by the QUENCHER procedure was shown to be higher or equivalent, than those obtained by sequential extraction [29].

### 3.4. Rheological Properties

#### 3.4.1. Small Amplitude Oscillatory Shear Measurements (SAOS)

**Figure 3:**
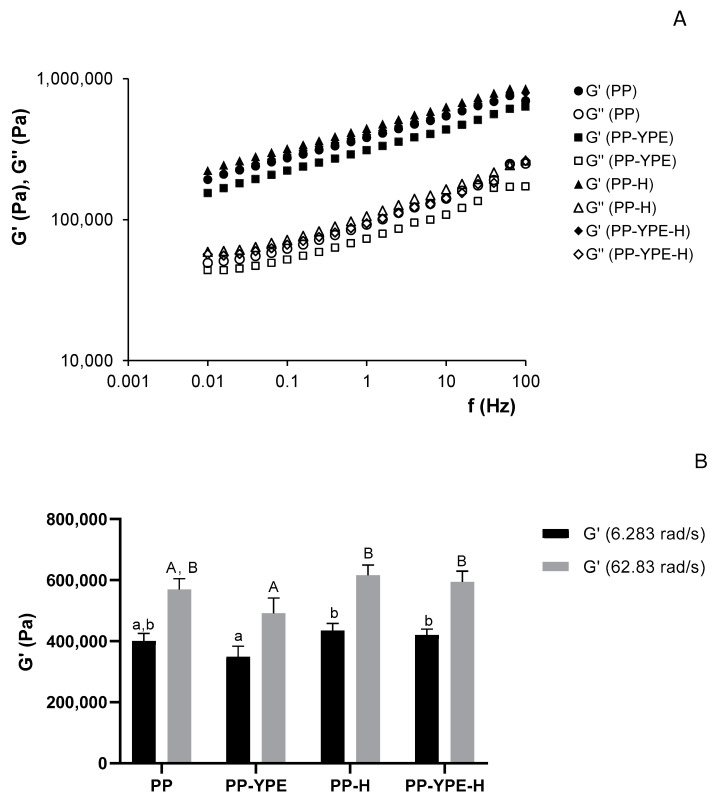
Mechanical spectra obtained for pear pomace (PP), pear pomace enriched with YPE (10% *w*/*w*) (PP-YPE), heated pear pomace (PP-H) and heated pear pomace enriched with YPE (10% *w*/*w*) (PP-YPE-H). G′ (storage modulus—filled symbol) and G′′ (loss modulus—open symbol) (**A**). Values of G′ at 1 Hz (6.283 rad/s) and 10 Hz (62.83 rad/s) obtained for each sample (**B**). Results are presented as mean ± standard deviation. Columns with different letters (uppercase or lowercase), are statistically different (*p* < 0.05).

The linear viscoelastic properties of the pear pomace pastes were examined using the dynamic oscillatory test. Frequency sweeps were conducted at constant shear stress, within the linear viscoelastic range, to investigate the degree of structure of pear pomace paste (PP). The impact of YPE addition (10% *w*/*w*) and thermal processing on pear pomace rheological behavior was also studied. For each sample, at least three individual replicates were analyzed, with the most representative mechanical spectra of each sample paste being presented on Figure 3**,** together with the G′ values at 1 Hz (PP, PP-YPE, PP-H and PP-YPE-H). The mechanical spectra of all formulations exhibited the typical behavior of soft gels, with G′ > G′′, for the whole range of frequencies studied, both demonstrating a slight dependence on the frequency and a destabilizing effect [33]. However, pear pomace enrichment with yeast protein extract (PP-YPE), revealed a slightly lower degree of structuring, expressed by lower values of viscoelastic functions (G′ and G′′), compared to the heated samples. For this sample, the elastic modulus value obtained at 1 Hz (G′ at 6.283 rad/s) was significantly lower (*p* < 0.05) than those for pear pomace and enriched samples subjected to thermal treatment (PP-H and PP-YPE-H). On the other hand, compared to control pear pomace paste (PP), the incorporation of 10% (*w*/*w*) of yeast protein did not cause significant differences in the paste structure (*p* > 0.05). Interestingly, thermal processing of YPE enriched pear pomace resulted on a significant structure recovery, to values close to the control (higher viscoelastic functions, G′ and G′′), possibly due to protein-starch matrix gelatinization (*p* > 0.05). These results seemed to indicate that protein-pear pomace interaction may have an impact on the properties of the paste, especially gelatinization process [43]. Comparing PP samples with or without thermal treatment, no significant differences could be observed. In fact, the real reinforcement of the structure should only be visible after heat treatment and cooling, when starch gelatinization may occur. Possibly due to low starch amount in pear pomace, this structure reinforcement was not significant with thermal processing.

From the power law equations, the frequency dependence of elastic (G′) and viscous modulus (G′′) could be observed [31], providing a good fit for the power law model, with high R^2^ values. By performing a linear regression on log G′ and G′′ vs. log frequency, the α′ and α′′ values together with β′ and β′′ values could be determined, corresponding to the y-intercepts and the slopes of the resulting line, respectively. As indicated in Table 3, and according to α′ and α′′ values, PP-YPE sample presented a significantly lower level of structure, compared to the other samples (*p* < 0.05), with 10% (*w*/*w*) protein fortification, inducing a destabilization of the structure network. This result is similar to others presented in the literature, describing a α′ and α′′ decrease, when higher amounts of fruit pomace were added. However, a structure recovery could be observed after thermal processing. During thermal treatment, proteins gelatinization should play an important role in pear pomace formulations, contributing to the structure restoring. Considering the present results, it is possible to state that YPE induces a destabilization of the network formed by pear pomace fibers [43].

**Figure 4:**
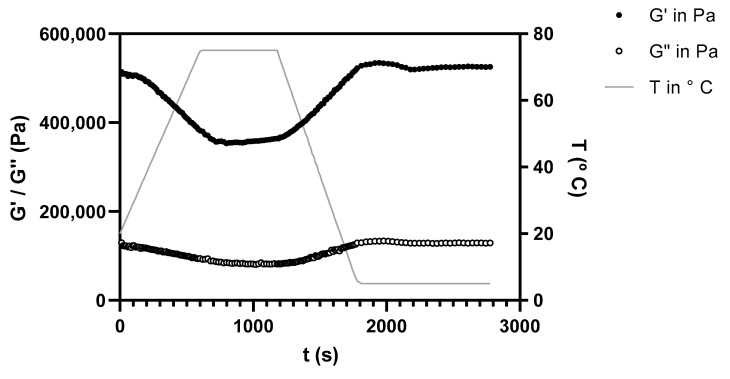
Evolution of storage modulus (G′) for pear pomace paste (PP) at 2π rad/s upon application of a thermal cycle (continuous line).

The evolution of yeast protein-enriched pear pomace (PP-YPE) SAOS’s properties, at 2π rad/s and over thermal treatment, is shown in Figure 4. The gel behavior was similar for both samples and may be described using four different stages: (i) the first region, simulating paste heating, displayed a slight decrease for G′ and G′′ values, related to some structure loss during heating and showing gel thermo reversibility, as some fiber or protein interconnection seemed to have been lost during heating (ii) the second region, corresponding to the thermal treatment at 75 °C, was characterized by a plateau for storage and loss moduli, reflecting gel stability after structuration; (iii) the third region, simulating the cooling stage, showed an increase in the viscoelastic properties, and the reinforcement of the gel network that had been loss during heating; (iv) the forth region was characterized by a new plateau, related to gel maturation. From these results, it could be observed that PP-YPE showed a structure that was temperature dependent, showing a structure loss during heating. On the other hand, these pastes also showed a quick and stable gel maturation after cooling.

#### 3.4.2. Apparent Viscosity

Pear pomace rheological behavior was studied before (PP) and after a heating step (PP-H) and with 10% YPE (*w*/*w*) enrichment (PP-YPE) (heated *vs* non-heated). The results regarding pastes flow behavior (Williamson’s model’ parameters), are presented on Table 4. All samples showed a similar shear-thinning behavior, presenting a constant viscosity at low shear rates (η_0_), followed by a continuous and gradual viscosity decrease with the shear rate increase. The obtained data for all tested pastes was fitted using the Williamson model, showing regression coefficients (R^2^) between 0.97 and 0.999, thus suggesting a good fit of the chosen model. All four samples showed high viscosity (approximately 10^6^ Pas.s), due to its high-water holding capacity, although this value being significantly lower for pear pomace enriched with YPE (*p* < 0.05), indicating a minor structuration level, as previously observed in the mechanical spectra. Additionally, YPE fortification may possibly reduce the water holding capacity of the paste, increasing the free water content and facilitating the particles movement, thus decreasing the apparent viscosity [44]. On the other hand, PP-YPE thermal processing caused a viscosity increase, indicating a higher resistance to flow and thus a structured recovery. For PP pastes, thermal treatment did not produce a viscosity difference big enough to be detected by the rheometer. Regarding the consistency index (k), heated samples presented a higher value, possibly due to the expected development of a stronger structure, but with no significant differences (*p* > 0.05). However, m values were significantly lower for the heated pastes (both pear pomace control and YPE-enriched pear pomace) (*p* < 0.05), thus suggesting the impact of heating on the pastes structure.

#### 3.4.3. Textural Parameters

**Figure 5:**
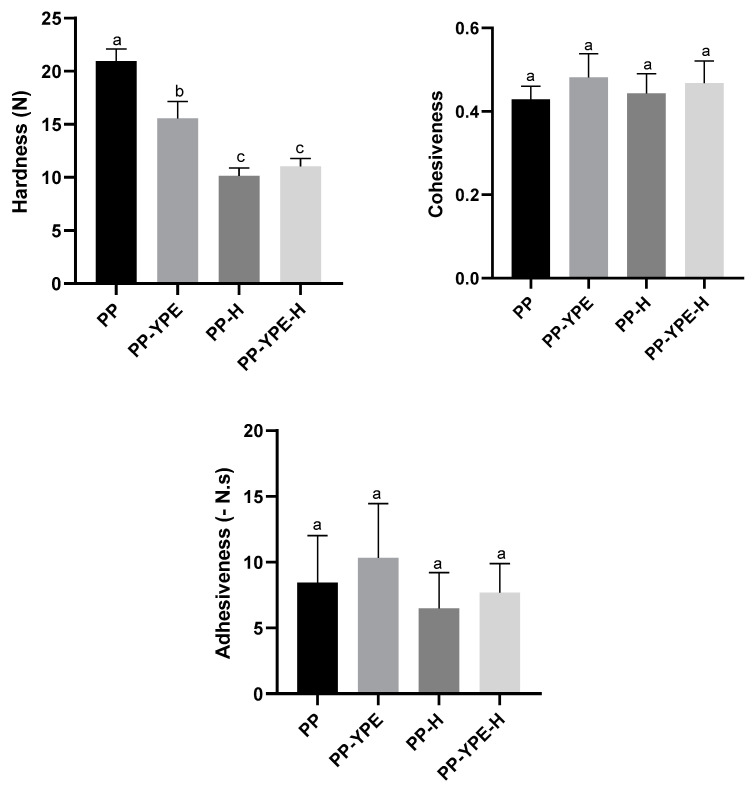
Hardness (N), cohesiveness and adhesiveness (-N.s) values of PP samples enriched with YPE (PP-YPE) and subjected to thermal treatment (PP-H and PP-YPE-H). Samples are presented as mean, with error bars indicating the standard deviations from the repetitions. In the same graph, different letters correspond to significant differences (*p* < 0.05).

Texture properties of all samples were assessed by a double penetration test, determining hardness, cohesiveness, and adhesiveness. These results are presented in Figure 5. The addition of YPE induced a significant decrease on sample’ hardness, in comparison to pear pomace sample (PP) (*p* < 0.05). This hardness decrease may arise from the structure weakening, due to yeast protein addition, as already observed from the viscoelastic measurements. On the other hand, after thermal treatment, an unexpected hardness decrease could also be observed, for both PP-H and PP-YPE-H, compared to non-heated samples (*p* < 0.05). According to the previously described rheological properties, due to the reinforcement of sample structure observed after heating treatment, an increase in hardness should also be expected, particularly for protein-enriched pomace. The water activity of s (a_w_) was also assessed and was found to be close to 0.98, with no significant differences between samples (*p* > 0.05). According to Ruiz-Ramírez et al. (2005), samples with similar a_w_ can also show important differences in hardness. On the other hand, samples with similar water content, can present similar hardness, with this indicating that hardness is more related to water content than to water activity [45]. During heat treatment, water content in the samples may have increased due to desorption from macromolecular hydrocolloids surface, with this resulting in a lower hardness. Regarding other textural parameters, such as cohesiveness and adhesiveness, no significant differences could be observed after YPE fortification or after thermal treatment.

#### 3.4.4. Microstructure of Pear Pomace Pastes

**Figure 6:**
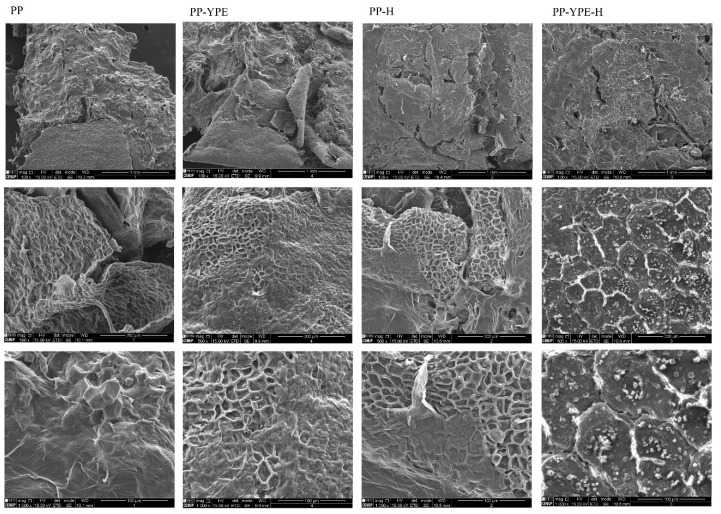
Scanning electron micrographs of PP samples enriched with YPE (PP-YPE) and subjected to thermal treatment (PP-H and PP-YPE-H). Samples are presented in three magnifications (from top to bottom: ×100; ×500 and ×1000).

Representative SEM micrographs of pear pomace samples with and without YPE or thermal treatment are shown in Figure 6 at three different magnification levels (100-, 500- and 1000-level). All samples microstructure presented a rough and irregular structure, making it possible to observe intact cell walls and fibrous structures. Pear pomace seemed to have the highest heterogeneity and roughness, presenting also a denser and more fibrous surface. On the other hand, PP-H and PP-YPE-H samples showed a more homogeneous and soft structure than those that were not subjected to thermal treatment, with these features being possibly correlated to the changes in the textural properties of these samples. Interestingly, protein enriched samples (PP-YPE and PP-YPE-H), showed the occurrence of a rather different microstructural feature. In fact, oval-shaped structures could be observed in these samples, with these shapes being particularly relevant for the heated sample (PP-YPE-H). These structures seemed to indicate a possible formation of protein aggregates, as well as to the entrapment of protein aggregates into the polysaccharides network, as previously described for egg yolk/kappa-carrageenan gels [46]. This moderate microstructural change is consistent with the significant increase observed for the linear viscoelastic properties of the pastes after thermal treatment, as these aggregates may have contributed for structural reinforcement, compared to the non-heated, enriched sample (PP-YPE).

## 4. Conclusions

The present work allowed us to characterize the composition, antioxidant capacity, rheological properties and microstructure of pear pomace, a co-product of the fruit juice industry. Pear pomace presented approximately 74% (DW) of dietary fibers and a strong antiradical capacity, due to the presence of free and bound phenolic compounds. The pear pomace showed a shear thinning and typical soft gel behavior, evidencing a potential application as a texturizing or thickening agent. Enrichment with yeast protein extract resulted in paste structure loss, and apparent viscosity reduction. However, when thermal processing was applied, a recovery of its rheological and textural properties could be observed. Thus, addition of YPE could increase fruit pomace protein content, without affecting its functional properties. Additionally, dietary fibers bound bioactive compounds, such as phenolic compounds, represent another health advantage of using this ingredient.

## Figures and Tables

**Table 1:** Composition of pear pomace (PP) and pear pomace enriched with YPE (PP-YPE).

	PP (Control)	PP-YPE (Mixture)
PP (g)	7.5	6.75
YPE (g)	-	0.75
Water (g)	15.00	15.00

**Table 2:** Proximate chemical composition of pear pomace flour, on a dry basis (% dry weight (DW)). Values are presented as means (*n* = 2).

	% (DW)
Moisture	6.3
Protein	1.8
Fat	1.1
Ash	1.1
Total Dietary Fibers (TDF)	74.5
Insoluble Dietary Fibers (IDF)	61
Soluble Dietary Fibers (SDF) *	13
Carbohydrates **	15.0

* Obtained by calculation ** Obtained as 100 − (fat + ash + protein + total dietary fiber).

**Table 3:** Frequency dependence of G′ and G′′ described by the power-law equations for pear pomace (PP), pear pomace enriched with YPE (10% *w/w*) (PP-YPE), heated pear pomace (PP-H) and heated pear pomace enriched with YPE (10% *w*/*w*) (PP-YPE-H). For each parameter samples with different letters are statistically different (*p* < 0.05). 0.9989 < R^2^ > 0.91.

	G′		G′′	
	α′	β′	α′′	β′′
PP	416,584 ± 33,245 ^a^	0.1445 ± 0.004 ^a^	105,220 ± 8601 ^a^	0.1773 ± 0.007 ^a^
PP-YPE	328,949 ± 43,319 ^b^	0.149 ± 0.003 ^a^	82,491 ± 11,109 ^b^	0.162 ± 0.006 ^b^
PP—H	428,146 ± 21,274 ^a^	0.148 ± 0.001 ^a^	107,692 ± 4453 ^a^	0.177 ± 0.006 ^a^
PP-YPE—H	402,475 ± 26,053 ^a^	0.147 ± 0.006 ^a^	100,326 ± 6825 ^a^	0.167 ± 0.005 ^a,b^

**Table 4:** Rheological parameters of the flow curves adjusted to the Williamson equation. Values are presented as mean ± standard deviation (*n* = 3). For each column, values with the same letter are not statistically different (*p* > 0.05).

	η_0_ (Pa.s)	m	k (s)	R^2^
PP	7.65 × 10^6^ ± 2.45 × 10^5 a^	1.28 ± 0.01 ^a^	795 ± 94 ^a^	0.99 ± 0.002
PP-YPE	5.94 × 10^6^ ± 4.15 × 10^5 b^	1.29 ± 0.01 ^a^	647 ± 81 ^a^	0.98 ± 0.03
PP—H	7.60 × 10^6^ ± 5.51 × 10^5 a^	1.17 ± 0.02 ^b^	1141 ± 599 ^a^	0.99 ± 0.003
PP-YPE—H	7.35 × 10^6^ ± 5.21 × 10^5 a^	1.20 ± 0.01 ^b^	1195 ± 379 ^a^	0.97 ± 0.02

## Supplementary Materials

The following supporting information can be downloaded at: https://www.mdpi.com/article/10.3390/molecules28010179/s1, Figure S1: HPLC−DAD/ESI-MS chromatogram of extractable phenolic fraction (EF) obtained from pear pomace. Peak assignment based on mass parent ion (*m/z*) and secondary (MS^2^ and MS^3^) fragment ions data. 1: quinic acid (*m/z* 191); 2: arbutin (*m/z* 271); 3: chlorogenic acid (*m/z* 353); 4: dimeric procyanidin (*m/z* 577); 5: caffeoylquinic acid (*m/z* 353); 6: (-)-epicatechin (*m/z* 289); 7: trimeric procyanidin (*m/z* 865); 8: n.d.; 9: quercetin-3-*O*-rutinoside (*m/z* 609); 10: quercetin-3-*O*-hexoside (*m/z* 463); 11: quercetin-3-*O*-galactoside (*m/z* 463); 12: isorhamnetin-3-*O*-rutinoside (*m/z* 623); Table S1: HPLC−DAD/ESI-MS profile of extractable phenolic fraction (EF) obtained from pear pomace.
