# Supplementary material for: Upcycling Rocha do Oeste Pear Pomace as a Sustainable Food Ingredient: Composition, Rheological Behavior and Microstructure Alone and Combined with Yeast Protein Extract
Source: Molecules. 2022 Dec 25;28(1):179. doi: 10.3390/molecules28010179 (PMC9822054; doi:10.3390/molecules28010179)
Supplement: Supplementary file 1 [file molecules-28-00179-s001.zip › molecules-2070515-supplementary.pdf]

Upcycling Rocha do Oeste pear pomace as a sustainable food ingredient: composition, rheological behavior and microstructure alone and combined with yeast protein extract

Ana Fernandes[1*], Sara Simões[2], Isabel. M. P. L. V. O. Ferreira[3], Maria João Alegria[4], Nuno Mateus[1], Anabela Raymundo[2], and Victor de Freitas[1]

[1] LAQV/REQUIMTE, Departamento de Química e Bioquímica, Faculdade de Ciências, Universidade do Porto, Porto, Portugal.

[2] LEAF - Linking Landscape, Environment, Agriculture and Food Research Centre, Instituto Superior de Agronomia da Universidade de Lisboa, Lisboa, Portugal.

[3]LAQV/REQUIMTE, Laboratory of Bromatology and Hydrology, Pharmacy Faculty, Porto University, Portugal.

[4] SUMOL+COMPAL Marcas S.A., Carnaxide, Portugal.

* Corresponding author. E-mail address: ana.fernandes@fc.up.pt (Ana Fernandes).

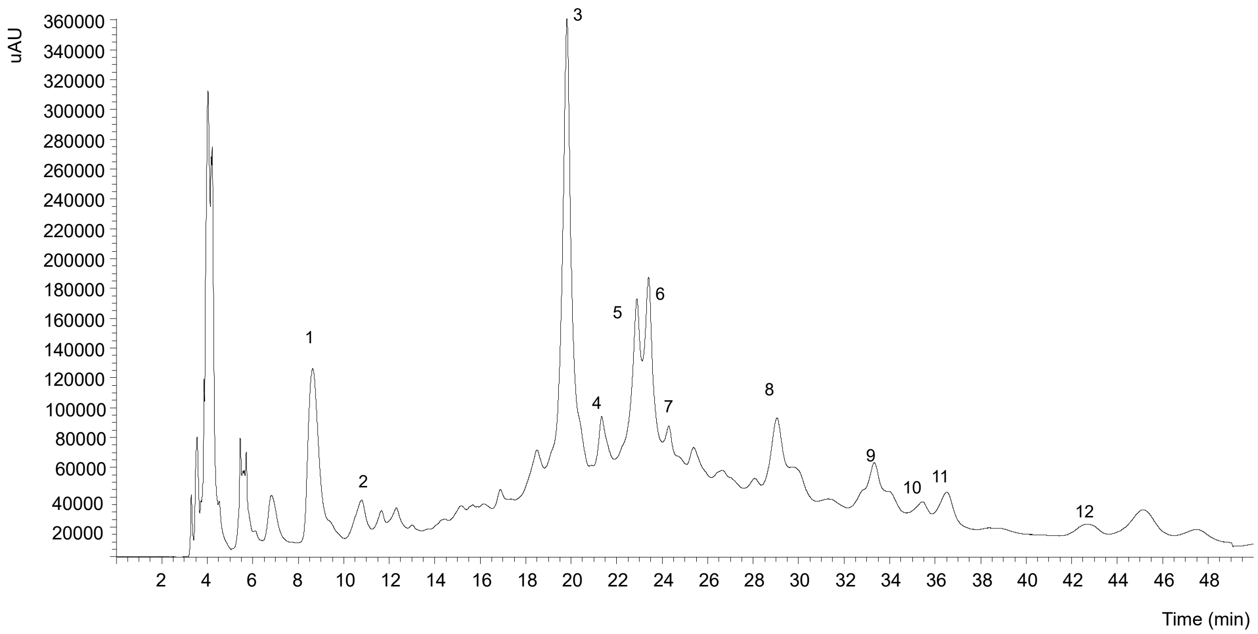

Figure S1. HPLC−DAD/ESI-MS chromatogram of extractable phenolic fraction (EF) obtained from pear pomace. Peak assignment based on mass parent ion (*m/z*) and secondary (MS^2 and MS^3) fragment ions data. 1: quinic acid (*m/z* 191); 2: arbutin (*m/z* 271); 3: chlorogenic acid (*m/z* 353); 4: dimeric procyanidin (*m/z* 577); 5: caffeoylquinic acid (*m/z* 353); 6: (-)-epicatechin (*m/z* 289); 7: trimeric procyanidin (*m/z* 865); 8: n.d.; 9: quercetin-3-*O*-rutinoside (*m/z* 609); 10: quercetin-3-*O*-hexoside (*m/z* 463); 11: quercetin-3-*O*-galactoside (*m/z* 463); 12: isorhamnetin-3-*O*-rutinoside (*m/z* 623).

Table S1. HPLC–DAD /ESI-MS profile of extractable phenolic fraction (EF) obtained from pear pomace.

Peak number	RT (min)	λ $_{máx}$ (nm)	$[M-H]^-$	MS^2	MS^3	Tentative identification
1	8.64	265	191.1	-	-	Quinic Acid
2	10.79	292	271.1	-	-	Arbutin
3	19.81	325	353.3	191.1	-	Chlorogenic acid
4	21.34	280	577.31	425.1; 407.2; 289.4	406.9	Dimeric procyanidin
5	22.88	283; 307	353.3	-	-	Caffeoyl-quinic acid
6	23.4	280	289.25	-	-	(-)-epicatechin
7	24.29	280	865.24	695; 577; 289	542.9; 451.1; 407	Trimeric procyanidin
8	29.04	268	739.04	586.73; 435; 289	407.1; 273.1	unknown
9	33.33	352	609.31	301.3	178.91; 271.1; 151	Quercetin-3-*O*-rutinoside
10	35.44	352	463.5	301.1; 179	179	Quercetin-3-*O*-hexoside
11	36.51	352	463.2	301.2	179.17; 151.1	Quercetin-3-*O*-galactoside
12	42.72	346	623.15	314.9; 300	300	Isorhamnetin-3-*O*-rutinoside
